# Risk Factors of Lymph Node Metastasis in Patients with Pancreatic Neuroendocrine Tumors (PNETs)

**DOI:** 10.1155/2020/1946156

**Published:** 2020-09-29

**Authors:** Zhe Wang, Feng Cao, Yupeng Zhang, Yu Fang, Fei Li

**Affiliations:** Department of General Surgery, Xuanwu Hospital, Capital Medical University, Beijing, China

## Abstract

**Background:**

The prognostic value of lymph node metastasis in patients with PNETs is controversial. Understanding the effect of lymph node metastasis on prognosis in pancreatic neuroendocrine tumors is helpful for surgery and follow-up. The purposes of this study are to identify predictors of lymph node metastasis among patients with PNETs and determine its prognostic associations.

**Methods:**

A retrospective analysis of the surveillance, epidemiology, and end results (SEER) database was performed. Patients with PNETs that underwent surgery and pathologic nodal staging were identified. Logistic regression and Cox regression were performed to identify independent predictors and prognostic factors, respectively.

**Results:**

Of 1956 patients (age: 56.8 ± 13.4 years, 53.3% males), 748 (38.2%) had lymph node metastasis. On multivariable analysis, tumor located in pancreas head, distant metastasis, and poorly differentiated, undifferentiated, and unknown differentiated histology grades were three independent risk factors of lymph node metastasis. In the entire cohort, lymph node metastasis indicated a worse overall survival (HR: 1.48, 95% CI: 1.17-1.88, *p* < 0.001) and disease-specific survival (HR: 1.87, 95% CI: 1.41-2.48, *p* < 0.001) on multivariable analysis. Lymph node metastasis was associated with worse overall (HR: 1.45, 95% CI: 1.08-1.93, *p* = 0.012) and disease-specific survival (HR: 2.13, 95% CI: 1.48-3.05, *p* < 0.001) in patients without distant metastasis on multivariate analysis. Lymph node metastasis was also independently associated with worse disease-specific survival among patients in well differentiation (HR: 2.16, 95% CI: 1.35-3.46, *p* = 0.001) and moderately differentiation (HR: 2.67, 95% CI: 1.28-5.56, *p* = 0.009) groups on multivariate analysis.

**Conclusions:**

Tumor located in pancreas head, distant metastasis, and poorly differentiated, undifferentiated, and unknown differentiated histology grades were three independent risk factors for lymph node metastasis. Lymph node metastasis was an independent prognostic factor of worse OS and DSS in patients with tumor located in pancreas head. Lymph node metastasis was an independent prognostic factor of worse OS and DSS in patients without distant metastasis. Lymph node metastasis was an independent prognostic factor of worse DSS in well differentiation and moderately differentiation groups.

## 1. Introduction

The neuroendocrine tumors (NETs) originate from neuroendocrine cells and may occur in many organs, including the lung, gastrointestinal tract, and pancreas. NETs occur in approximately 6/100,000 [1]. Among them, gastroenteropancreatic NETs (GEP-NETs) account for 65-75% of the whole body NETs. Pancreatic NETs (PNETs) comprise approximately half of the GEP-NETs [2] and account for approximately <3% of all pancreatic malignancies. As a rare pancreatic neoplasm with an annual incidence of 0.19/100,000-0.32/100,000 [3], the incidence of PNETs has been rising in the United States over the past several decades [4].

The natural history of PNETs is highly variable, with some tumors showing indolent behavior but others displaying an aggressive course, with local invasion and distant metastasis [5]. Several staging systems have been developed to better stratify prognosis in patients with PNETs. The World Health Organization staging system incorporates mitotic count and Ki-67 index to separate patients into three categories [6]. The eighth edition of the American Joint Committee on Cancer (AJCC) tumor–node–metastasis staging system includes primary tumor size, presence of lymph node metastasis on pathologic examination, and presence of distant metastases [7]. Lymph node status is particularly important in PNETs staging systems, because the presence of lymph node metastasis would render a patient stage III according to both the AJCC and European Neuroendocrine Tumor Society staging (ENETS) systems [8]. The prognostic significance of lymph node metastasis in PNETs is controversial. Some researchers have reported a significant association between lymph node metastasis and survival [9–15], while others showed no association [16–19]. Lymph node metastasis is an important marker of malignancy and, as such, may influence the type and extent of PNETs surgical management. The purposes of this study are to identify predictors of lymph node metastasis among patients with PNETs and determine its prognostic associations.

## 2. Methods

**Figure 1:**
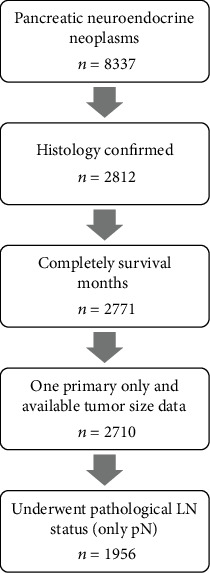
Flowchart of the patients selection process. A total of 8337 cases of pancreatic neuroendocrine neoplasms (PNEN) from SEER database were screened and 1956 cases were included in the final analysis.

A retrospective analysis of the National Cancer Institute's SEER database was performed for patient diagnosed between 2004 and 2016. The SEER database is a comprehensive database that collects information on several clinical and pathologic aspects of multiple cancers and is approximated to encompass 28% of the US population. Pancreatic neuroendocrine tumors were identified using their ICD-O-3 codes (8013/3, 8150/3, 8151/3, 8152/3, 8153/3, 8155/3, 8156/3, 8240/3, 8241/3, 8242/3, 8243/3, 8245/3, 8246/3, 8247/3, 8248/3, and 8249/3). Patients without positive histological diagnosis and those that did not undergo surgical resection were excluded. Patients without available information regarding their lymph node status were also excluded. Only patients that met criteria for pathologic nodal staging (PN staging) were included based on the SEER variable CS lymph Nodes Eval (CS Lymph Nodes Eval code 3 and code 6). In this way, patients without pathological nodal staging were excluded from the analysis. A flowchart demonstrating patient selection can be viewed in Figure 1. All tumors were primary tumors. The study was exempted from Institutional Review Board approval, due to SEER's use of unidentifiable patient information.

## 3. Statistical Analysis

Univariate and multivariable logistic regressions were performed to ascertain the possible factors associated with the presence of lymph node metastasis. Univariate and multivariable analyses using the Cox proportional hazards model were performed to ascertain the prognostic role of nodal metastasis, with both overall survival (OS) and disease-specific survival (DSS) as endpoints. All statistical tests used two-tailed *p* values, and 0.05 was set as the threshold for significance. Statistical analysis was performed using the IBM SPSS Statistics v.22.0.0 (IBM Corp, Armonk, NY).

## 4. Results

Overall, 1956 patients were identified, of which 748 patients (38.2%) had lymph node metastasis. The median number of lymph node examined was 10 (mean 12.8 ± 8.5), and the median number of metastatic lymph nodes was 2 (mean 3.5 ± 3.8). Most tumors were located in the body and tail of pancreas (1016 (51.9%)), followed by the head of pancreas (632 (32.3%)) and the overlapping lesion and other specified parts of pancreas (308 (15.7%)). Most patients were males (1043 (53.3%)) and 301 (15.4%) patients had synchronous distant metastasis at diagnosis. Patients were followed for a median period of 49.1 months (range 0-155 months). Detailed demographic, clinical, and pathologic features of the study cohort are listed in Table 1.

### 4.1. Risk Factors of Lymph Node Metastasis

On univariate analysis, race (*p* = 0.034), tumor location (*p* < 0.001), tumor size (*p* = 0.008), M stage of the disease (*p* < 0.001), and histology grade (*p* < 0.001) were associated with the presence of lymph node metastasis. These variables were then included in multivariable analysis. Finally, patients with tumor located in pancreas head, distant metastasis, and poorly differentiated, undifferentiated, and unknown differentiated histology grades were three independent risk factors associated with the diagnosis of the patient lymph node metastasis (Table 2).

### 4.2. Prognostic Value of Lymph Node Metastasis

Seven hundred and forty-eight patients with lymph node metastasis had a median overall survival of 45 months, while median overall survival was 41 months in patients without lymph node metastasis. Excluding 301 (15.4%) patients with distant metastasis, the median overall survival time for remaining 1655 (84.6%) patients with lymph node metastasis was 42 months, and the median overall survival of patients without lymph node metastasis was 40 months.

On univariate analysis, lymph node metastasis (51.7 ± 1.3 vs. 47.5 ± 0.9 months, *p* < 0.001), older age (<40 years: 52.5 ± 2.4 months, 40-59 years, 52.5 ± 1.2 months, 60-79 years: 45.2 ± 1.1 months, and ≥80 years: 40.7 ± 4.2 months, *p* < 0.001), male (47.2 ± 1.0 vs. 51.2 ± 1.2 months, *p* = 0.006), unmarried (unmarried 46.9 ± 1.4 months, married: 50.2 ± 1.0 months, *p* = 0.022), uninsured (uninsured: 42.3 ± 3.9 months, insured: 44.6 ± 0.7 months, *p* < 0.001), tumor located in the pancreas head (head: 50.1 ± 1.5 months, body and tail: 47.4 ± 1.0 months, and overlapping: 46.3 ± 2.8 months, *p* = 0.006), large tumor size (<2 cm: 45.4 ± 1.3 months, 2-4 cm: 50.1 ± 1.3 months, and >4 cm: 50.8 ± 1.3 months, *p* < 0.001), distant metastasis (45.7 ± 1.9 vs. 49.6 ± 0.8 months, *p* < 0.001), and lower levels of differentiation (well differentiated: 47.6 ± 0.9 months, moderately differentiated: 43.2 ± 1.7 months, poorly differentiated: 37.9 ± 3.2 months, and undifferentiated: 26.5 ± 6.1 months, *p* < 0.001) were associated with worse OS. While, on multivariable analysis lymph node metastasis was still an independent factor indicated worse OS (HR: 1.48, 95% CI: 1.17-1.88, *p* = 0.001) (Table 3).

**Figure 2:**
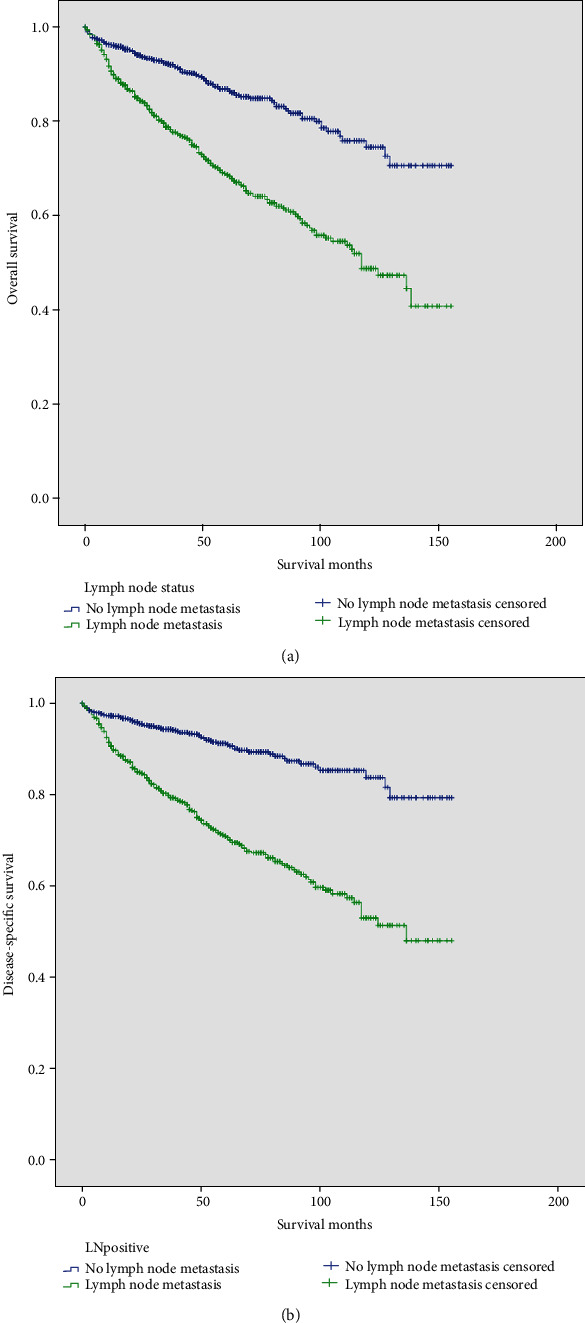
The Kaplan-Meier curve depicting the overall survival (a) and disease-specific survival (b) of patients with and without lymph node metastasis.

On univariate analysis, lymph node metastasis (52.0 ± 1.3 vs. 47.9 ± 0.9 months, *p* < 0.001), older age (<40 years: 52.4 ± 2.5 months, 40-59 years, 52.7 ± 1.2 months, 60-79 years: 45.8 ± 1.1 months, and ≥80 years: 40.8 ± 4.4 months, *p* = 0.046), male (47.7 ± 1.0 vs. 51.5 ± 1.2 months, *p* = 0.014), unmarried (unmarried 46.8 ± 1.4 months, married: 50.8 ± 1.0 months, *p* = 0.016), uninsured (uninsured: 41.7 ± 3.8 months, insured: 45.1 ± 0.7 months, *p* < 0.001), tumor located in the pancreas head (head: 50.4 ± 1.5 months, body and tail: 47.8 ± 1.0 months, and overlapping: 47.5 ± 2.8 months, *p* = 0.011), large tumor size (<2 cm: 45.5 ± 1.3 months, 2-4 cm: 50.4 ± 1.3 months, and >4 cm: 51.6 ± 1.3 months, *p* < 0.001), distant metastasis (45.9 ± 1.9 vs. 50.0 ± 0.9 months, *p* < 0.001), and lower levels of differentiation (well differentiated: 48.1 ± 0.9 months, moderately differentiated: 43.8 ± 1.7 months, poorly differentiated: 37.5 ± 3.2 months, and undifferentiated: 25.6 ± 6.3 months, *p* < 0.001) were associated with worse DSS. However, on multivariable analysis, lymph node metastasis was also an independent factor indicated worse DSS (HR: 1.87, 95% CI: 1.41-2.48, *p* < 0.001) (Table 4; Figure 2).

Among patients with lymph node metastasis, a higher number of positive lymph nodes was related to worse OS (HR 1.01, 95% CI: 1.00-1.02, *p* = 0.017) and DSS (HR 1.01, 95% CI: 0.99-1.02, *p* = 0.070) on univariate analysis.

### 4.3. Subgroup Analysis of Tumor Location, Distant Metastasis, and Histology Grade

Subgroup analysis of tumor location, distant metastasis, and histology grade were executed to ascertain whether the diagnosis of lymph node metastasis was independent prognostic factor in these groups of patients.

According to the tumor location, 632 (32.3%) patients have their tumor located in the pancreas head. Among this subgroup, 295 (46.7%) patients had lymph node metastasis. On univariate analysis, lymph node metastasis was related to both worse OS (HR: 2.46, 95% CI: 1.73-3.51, *p* < 0.001) and DSS (HR: 2.91, 95% CI: 1.93-4.40, *p* < 0.001). Also, the result was worse OS (HR: 1.83, 95% CI: 1.23-2.70, *p* = 0.003) and DSS (HR: 2.03, 95% CI: 1.29-3.22, *p* = 0.002) on multivariate analysis. Among the 1324 (67.7%) patients whose tumor is located in the pancreas body, tail, overlapping, and other parts, lymph node metastasis was related to worse OS (HR: 2.48, 95% CI: 1.88-3.28, *p* < 0.001) and DSS (HR: 3.97, 95% CI: 2.83-5.55, *p* < 0.001) on univariate analysis. And, the result was worse OS (HR: 1.36, 95% CI: 0.99-1.87, *p* = 0.046) and DSS (HR: 1.92, 95% CI: 1.32-2.80, *p* = 0.001) on multivariate analysis.

In the subgroup analysis of 301 (15.4%) patients with distant metastasis, lymph node metastasis was related to worse OS (HR: 1.52, 95% CI: 0.99-2.33, *p* = 0.045) but did not include worse DSS (HR: 1.41, 95% CI: 0.92-2.16, *p* = 0.120) on univariate analysis. The result of OS (HR: 1.54, 95% CI: 0.98-2.41, *p* = 0.060) and DSS (HR: 1.42, 95% CI: 0.90-2.24, *p* = 0.129) on multivariate analysis was not statistically significant. Among 1644 (84.0%) patients without distant metastasis and 11 (0.6%) patients with unknown metastasis status, lymph node metastasis was related to both worse OS (HR: 2.15, 95% CI: 1.65-2.81, *p* < 0.001) and DSS (HR: 3.52, 95% CI: 2.52-4.92, *p* < 0.001) on univariate analysis. And the result was worse OS (HR: 1.45, 95% CI: 1.08-1.93, *p* = 0.012) and DSS (HR: 2.13, 95% CI: 1.48-3.05, *p* < 0.001) on multivariate analysis.

Consider the histology grade, there were 1234 (63.1%) patients with well-differentiated, 319 (16.3%) patients with moderately differentiated, 109 (5.6%) patients with poorly differentiated, and 24 (1.2%) patients with undifferentiated histology grades. In the well-differentiated group and moderately differentiated group, lymph node metastasis was related to both worse OS (well-differentiated group HR: 1.96, 95% CI: 1.41-2.73, *p* < 0.001; moderately differentiated group HR: 2.13, 95% CI: 1.19-3.83, *p* = 0.011) and DSS (well-differentiated group HR: 3.46, 95% CI: 2.27-5.29, *p* < 0.001; moderately differentiated group HR: 2.92, 95% CI: 1.49-5.69, *p* = 0.002) on univariate analysis. However, in poorly differentiated group and undifferentiated group, the OS (poorly differentiated group HR: 1.74, 95% CI: 0.91-3.36, *p* = 0.095; undifferentiated group HR: 1.72, 95% CI: 0.46-6.46, *p* = 0.425) and DSS (poorly differentiated group HR: 2.04, 95% CI: 1.00-4.16, *p* = 0.149; undifferentiated group HR: 2.22, 95% CI: 0.48-10.19, *p* = 0.305) were not statistically significant. On multivariate analysis, there was no statistically significant difference in OS (well-differentiated group HR: 1.33, 95% CI: 0.92-1.91, *p* = 0.131; moderately differentiated group HR: 1.75, 95% CI: 0.93-3.31, *p* = 0.085; poorly differentiated group HR: 1.93, 95% CI: 0.91-4.09, *p* = 0.086; and undifferentiated group HR: 0.89, 95% CI: 0.01-7.46, *p* = 0.284) in each differentiated subgroup. In addition, lymph node metastasis was related to worse DSS in the well-differentiated group (HR: 2.16, 95% CI: 1.35-3.46, *p* = 0.001) and moderately differentiated group (HR: 2.67, 95% CI: 1.28-5.56, *p* = 0.009) on multivariate analysis. Whereas, the DSS in poorly differentiated group (HR: 2.30, 95% CI: 1.01-5.23, *p* = 0.064) and undifferentiated group (HR: 0.09, 95% CI: 0.01-7.48, *p* = 0.285) did not achieve statistical significance on multivariate analysis.

## 5. Discussion

This study demonstrated that tumor located in pancreas head, distant metastasis, and poorly differentiated, undifferentiated, and unknown differentiated histology grades were three independent risk factors associated with the diagnosis of lymph node metastasis in patients with PNETs, suggesting that a high degree of suspicion for lymph node metastasis should be present in these patients. Lymph node metastasis was also identified as an independent predictor of worse overall and disease-specific survival and in patients with PNETs. And lymph node metastasis was an independent prognostic factor of worse overall survival and disease-specific survival in patients without distant metastasis. Furthermore, lymph node metastasis was an independent prognostic factor of worse disease-specific survival in patients with the well-differentiated and moderately differentiated groups.

Data concerning the incidence of lymph node metastasis in patients with PNETs is multifold. Hill and colleagues retrospectively analyzed 728 patients with PNETs and identified lymph node metastasis in only 24.9% using the SEER database [20]. The rate of lymph node metastasis that was identified in our study was 38.7%, which is in line with previously published data by Hashim and colleagues, who reported a rate of 37.6% [5]. Moreover, a 33.3% rate of lymph node metastasis was reported by the PET/CT screening imaging [21]. However, our rate of 38.7% may be an overestimation of the true incidence of lymph node metastasis due to the exclusion of low-risk patients that did not undergo nodal sampling due to a low degree of suspicion.

This study identified tumor located in pancreas head, distant metastasis, and poorly differentiated, undifferentiated, and unknown differentiated histology grades as three independent predictors of lymph node metastasis in patients with PNETs. As is well known, pancreatic resections are associated with significant morbidity, and there is interest in minimizing the impact of surgery [22]. Hence, enucleation and central pancreatectomy in selected patients, especially in those with small tumor size and tumor not close to the main pancreatic duct, could be considered as alternative management strategies to radical surgery. For instance, Tsutsumi and colleagues suggested that regional lymphadenectomy should be performed for all PNETs ≥ 15 mm [23], while Jiang and colleagues suggested that regional lymphadenectomy in PNETs ≤ 25 mm is not necessary [24]. Curran and colleagues found there was no lymph node metastasis in patients with PNETs ≤ 1 cm in low tumor grade, so they suggested regional lymphadenectomy is unnecessary in these patients [25].

However, assessing lymph node status preoperatively by radiological examination or endoscopy cannot be accurate and facile; preoperative determination of the risk of lymph node metastases attains critical importance. Therefore, patients without known lymph node status but fulfilling these factors should be considered for pancreatectomy with regional lymphadenectomy instead of tumor enucleation or central pancreatectomy without nodal staging.

The results of this study also demonstrated that lymph node metastasis was correlated with worse OS and DSS on multivariate analysis. Moreover, the number of more lymph nodes metastasis has a certain influence on OS and DSS, which suggests that standard regional lymphadenectomy may guide accurate staging, thus having a good influence on the prognosis.

The subgroup analysis results of multivariate analysis by tumor location grouping were also worse OS and DSS. The OS and DSS were statistically significant only in PNETs patients without distant metastasis, in either univariate or multivariate analyses. This suggested that lymphadenectomy is not necessary in PNETs patients with distant metastasis. On multivariate analysis based on the grouping of tumor differentiation grade, there was no significant difference in OS among each subgroup. However, OSS only showed significant difference between the well differentiation group and the moderately differentiation group. The effect of lymph node metastasis to prognosis is controversial in patients with PNETs. Some reports have associated lymph node metastasis with a shorter OS and DSS [9–15]. However, several other studies have reported that lymph node metastasis has no effect on survival [16–19]. It is difficult to interpret the real reasons of the contradictory results reported by different institutions. The inconsistency may be in part due to inadequate lymph node sampling rates during pancreatic resections and various measured outcome (OS, 5-year OS, DFS, DSS, etc.). Therefore, we interpreted this data to recommend that regional lymphadenectomy may be a safer choice for even low-grade patients. Meanwhile, lymph node metastasis is related to pathological features such as lymphatic, vascular, and neural invasion [26]. So, it is possible that with the extension of follow-up time, patients with high-grade PNETs will recurrence and die, so OS and DSS will become statistically significant.

There were certain several limitations for this study. The design of the study was retrospective. Large databases contain patients from different institutions and time periods. This can lead to patient selection bias, coding ,errors and missing information. Other variables, such as performance status, comorbidities, mitotic count, Ki-67 index, and surgical information (duration, blood loss, and postoperative complication), were not captured in the SEER database. In addition, there was no information on whether adjuvant therapies (chemotherapy, targeted therapy, and endocrine therapy) were given to patients with PNETs, which may have contributed to a better analysis. Despite these limitations, large database like SEER is the best database available today. We are in agreement of prospective study to better understand the risk factors of lymph node metastasis in PNETs and their effects on prognosis through the inclusion of more impact factors.

## 6. Conclusion

In conclusion, this study was performed on PNETs patients who underwent surgical treatment in the SEER database. The research results confirmed that tumor located in pancreas head, distant metastasis, and poorly differentiated, undifferentiated, and unknown differentiated histology grades were three independent risk factors for lymph node metastasis. This suggested that regional lymphadenectomy should be carefully considered when choosing surgical treatment for these patients. In addition, lymph node metastasis is an independent prognostic factor of worse OS and DSS in patients with tumor located in the pancreas head. Lymph node metastasis was an independent prognostic factor of worse OS and DSS in patients without distant metastasis. However, lymph node metastasis was an independent prognostic factor for worse DSS in well differentiation and moderately differentiation groups. Prospective studies are required to more comprehensively understand the risk factors of lymph node metastasis and determine criteria in performing regional lymphadenectomy in patients with PNETs.

## Figures and Tables

**Table 1:** Detailed demographic, clinical, and pathological features of the study cohort.

	No. of patients (*n* = 1956)	Patients with LN metastases (*n* = 748)
Age at diagnosis	56.83 ± 13.39 years	56.36 ± 13.39 years
Sex		
Male	1043 (53.3%)	401 (53.6%)
Female	913 (46.7%)	347 (46.4%)
Race		
White	1289 (65.9%)	510 (68.2%)
Black	223 (11.4%)	94 (12.6%)
Hispanic	249 (12.7%)	85 (11.4%)
Asian	168 (8.6%)	51 (6.8%)
Other	27 (1.4%)	8 (1.1%)
Marital status		
Unmarried	607 (31.0%)	233 (31.1%)
Married	1251 (64.0%)	482 (64.4%)
Unknown	98 (5.0%)	33 (4.4%)
Insurance status		
Uninsured	47 (2.4%)	17 (2.3%)
Insured	1689 (86.3%)	619 (82.8%)
Unknown	220 (11.2%)	112 (15.0%)
Tumor location		
Head	632 (32.3%)	295 (39.4%)
Body and tail	1016 (51.9%)	332 (44.4%)
Overlapping	111 (5.7%)	46 (6.1%)
Other	197 (10.1%)	75 (10.0%)
Tumor size		
<2 cm	526 (26.9%)	75 (10.0%)
2 cm-4 cm	708 (36.2%)	283 (37.8%)
>4 cm	718 (36.7%)	389 (52.0%)
Unknown	4 (0.2%)	1 (0.1%)
M stage		
M0	1644 (84.0%)	521 (69.7%)
M1	301 (15.4%)	221 (29.5%)
Mx	11 (0.6%)	6 (0.8%)
Histology grade^a^		
Well differentiated	1234 (63.1%)	401 (53.6%)
Moderately differentiated	319 (16.3%)	127 (17.0%)
Poorly differentiated	109 (5.6%)	84 (11.2%)
Undifferentiated	24 (1.2%)	18 (2.4%)
Unknown	270 (13.8%)	118 (15.8%)

^a^Histology grade was based on WHO classification of tumors 4th edition.

**Table 2:** Results of univariate and multivariable analysis for the possible factors of lymph node metastasis.

	Univariate		Multivariable	
	OR (95% CI)	*p* value	Adjusted OR (95% CI)	*p* value
Age^a^	0.99 (0.98-1.01)	0.163		
Sex		0.867		
Male	Reference			
Female	1.02 (0.83-1.25)			
Race		0.034		0.058
White	Reference		Reference	
Black	1.24 (0.91-1.70)		1.28 (0.92-1.78)	0.144
Hispanic	0.80 (0.59-1.10)		0.75 (0.55-1.04)	0.081
Asian	0.64 (0.44-0.94)		0.63 (0.42-0.92)	0.018
Other	0.67 (0.28-1.62)		0.80 (0.32-2.00)	0.629
Marital status		0.420		
Unmarried	Reference			
Married	1.06 (0.85-1.33)			
Unknown	0.78 (0.48-1.28)			
Insurance status		0.165		
Uninsured	Reference			
Insured	1.33 (0.68-2.57)			
Unknown	1.74 (0.85-3.58)			
Tumor location		<0.001		<0.001
Head	Reference		Reference	
Body and tail	0.54 (0.43-0.67)		0.54 (0.43-0.68)	<0.001
Overlapping	0.69 (0.43-1.08)		0.70 (0.44-1.13)	0.144
Other	0.68 (0.48-0.97)		0.69 (0.48-0.99)	0.045
Tumor size^a^	1.01 (1.00-1.05)	0.008	1.03 (1.00-1.06)	0.347
M stage		<0.001		<0.001
M0	Reference		Reference	
M1	5.15 (3.85-6.90)		4.09 (3.04-5.51)	<0.001
Mx	2.77 (0.80-9.59)		2.31 (0.65-8.25)	0.197
Histology grade		<0.001		<0.001
Well differentiated	Reference		Reference	
Moderately differentiated	1.11 (0.84-1.46)		0.95 (0.72-1.26)	0.728
Poorly differentiated	5.12 (3.14-8.35)		3.90 (2.39-6.36)	<0.001
Undifferentiated	3.84 (1.43-10.33)		4.18 (1.51-11.57)	0.006
Unknown	1.33 (0.98-1.79)		1.38 (1.01-1.87)	0.041

^a^Continuous variables.

**Table 3:** Results of univariate and multivariable analysis for overall survival in the entire study cohort.

	Univariate		Multivariable	
	HR (95% CI)	*p* value	Adjusted HR (95% CI)	*p* value
Age		<0.001		<0.001
<40	Reference		Reference	
40-59	1.42 (0.94-2.15)	0.042	1.37 (0.90-2.08)	0.014
60-79	1.84 (1.22-2.77)	0.003	2.06 (1.36-3.12)	0.001
≥80	3.48 (1.92-6.33)	<0.001	3.30 (1.79-6.06)	<0.001
Sex		0.006		0.003
Male	Reference		Reference	
Female	0.74 (0.60-0.92)	0.006	0.71 (0.57-0.89)	0.003
Race		0.451		
White	Reference			
Black	0.92 (0.65-1.30)	0.634		
Hispanic	0.90 (0.64-1.26)	0.523		
Asian	0.68 (0.43-1.06)	0.086		
Other	0.65 (0.21-2.03)	0.456		
Marital status		0.022		0.050
Unmarried	Reference		Reference	
Married	0.86 (0.68-1.07)	0.017	0.76 (0.60-0.96)	0.022
Unknown	0.66 (0.37-1.17)	0.016	0.65 (0.37-1.17)	0.149
Insurance status		<0.001		0.005
Uninsured	Reference		Reference	
Insured	0.53 (0.30-0.95)	0.033	0.51 (0.28-0.92)	0.026
Unknown	0.92 (0.50-1.70)	0.080	0.73 (0.39-1.36)	0.318
Tumor location		0.006		0.034
Head	Reference		Reference	
Body and tail	0.67 (0.53-0.84)	0.001	0.71 (0.56-0.90)	0.005
Overlapping	0.95 (0.61-1.49)	0.824	0.78 (0.49-1.23)	0.284
Other	0.81 (0.57-1.15)	0.234	0.97 (0.68-1.39)	0.871
Tumor size		<0.001		0.005
<2 cm	Reference		Reference	
2 cm-4 cm	2.38 (1.63-3.47)	<0.001	1.72 (1.16-2.53)	0.007
>4 cm	3.61 (2.52-5.17)	<0.001	2.01 (1.37-2.96)	<0.001
Unknown		0.999		0.941
M stage		<0.001		<0.001
M0	Reference		Reference	
M1	3.58 (2.86-4.45)	<0.001	2.61 (2.06-3.31)	<0.001
Mx	0.98 (0.24-3.96)	0.979	0.87 (0.21-3.55)	0.849
Histology grade		<0.001		<0.001
Well differentiated	Reference		Reference	
Moderately differentiated	1.41 (1.01-1.95)	0.401	1.13 (0.81-1.57)	0.474
Poorly differentiated	6.07 (4.51-8.16)	<0.001	3.56 (2.62-4.84)	<0.001
Undifferentiated	7.81 (4.42-13.81)	<0.001	5.80 (3.22-10.45)	<0.001
Unknown	1.78 (1.35-2.35)	<0.001	1.46 (1.10-1.95)	0.010
Lymph node metastasis		<0.001		<0.001
No metastasis	Reference		Reference	
Metastasis	2.55 (2.05-3.17)	<0.001	1.48 (1.17-1.88)	0.001

**Table 4:** Results of univariate and multivariable analysis for disease-specific survival in the entire study cohort.

	Univariate		Multivariable	
	HR (95% CI)	*p* value	Adjusted HR (95% CI)	*p* value
Age		0.046		0.012
<40	Reference		Reference	
40-59	1.31 (0.85-2.03)	0.226	1.27 (0.81-1.99)	0.303
60-79	1.54 (0.99-2.38)	0.056	1.76 (1.12-2.75)	0.013
≥80	2.38 (1.16-4.85)	0.018	2.16 (1.04-4.48)	0.038
Sex		0.014		0.009
Male	Reference		Reference	
Female	0.74 (0.58-0.94)	0.014	0.69 (0.54-0.89)	0.004
Race		0.584		
White	Reference			
Black	0.99 (0.68-1.44)	0.962		
Hispanic	0.81 (0.54-1.21)	0.306		
Asian	0.70 (0.43-1.15)	0.162		
Other	0.84 (0.27-2.61)	0.757		
Marital status		0.016		0.040
Unmarried	Reference		Reference	
Married	0.81 (0.63-1.04)	0.010	0.72 (0.55-0.94)	0.016
Unknown	0.63 (0.33-1.20)	0.016	0.62 (0.32-1.21)	0.163
Insurance status		<0.001		0.007
Uninsured	Reference		Reference	
Insured	0.54 (0.28-1.06)	0.075	0.52 (0.26-1.04)	0.065
Unknown	1.08 (0.54-2.16)	0.836	0.80 (0.39-1.63)	0.533
Tumor location		0.011		0.064
Head	Reference		Reference	
Body and tail	0.69 (0.50-0.84)	0.001	0.71 (0.54-0.93)	0.012
Overlapping	0.98 (0.59-1.61)	0.925	0.81 (0.49-1.36)	0.429
Other	0.79 (0.53-1.18)	0.254	1.01 (0.67-1.53)	0.951
Tumor size		<0.001		0.014
<2 cm	Reference		Reference	
2 cm-4 cm	3.05 (1.90-4.90)	<0.001	1.90 (1.17-3.11)	0.010
>4 cm	5.02 (3.18-7.91)	<0.001	2.23 (1.37-3.62)	0.001
Unknown		0.948		0.940
M stage		<0.001		<0.001
M0	Reference		Reference	
M1	4.89 (3.85-6.21)	<0.001	3.21 (2.47-4.16)	<0.001
Mx	1.44 (0.36-5.81)	0.611	1.21 (0.30-4.95)	0.792
Histology grade		<0.001		<0.001
Well differentiated	Reference		Reference	
Moderately differentiated	1.73 (1.20-2.50)	0.064	1.32 (0.91-1.92)	0.144
Poorly differentiated	8.45 (6.11-11.68)	<0.001	4.64 (3.31-6.49)	<0.001
Undifferentiated	10.61 (5.81-19.39)	<0.001	7.65 (4.10-14.27)	<0.001
Unknown	2.15 (1.56-2.95)	<0.001	1.69 (1.21-2.36)	0.002
Lymph node metastasis		<0.001		<0.001
No metastasis	Reference		Reference	
Metastasis	3.63 (2.80-4.72)	<0.001	1.87 (1.41-2.48)	<0.001

## Data Availability

The data used to support the findings of the study “Risk factors of lymph node metastasis in patients with pancreatic neuroendocrine tumors (PNETs)” have been deposited in the SEER database. The method of obtaining data has been described in detail in the manuscript.
